# Proteasome Nuclear Activity Affects Chromosome Stability by Controlling the Turnover of Mms22, a Protein Important for DNA Repair

**DOI:** 10.1371/journal.pgen.1000852

**Published:** 2010-02-19

**Authors:** Shay Ben-Aroya, Neta Agmon, Karen Yuen, Teresa Kwok, Kirk McManus, Martin Kupiec, Philip Hieter

**Affiliations:** 1 Michael Smith Laboratories, University of British Columbia, Vancouver, Canada; 2 Department of Molecular Microbiology and Biotechnology, Tel Aviv University, Ramat Aviv, Israel; National Cancer Institute, United States of America

## Abstract

To expand the known spectrum of genes that maintain genome stability, we screened a recently released collection of temperature sensitive (Ts) yeast mutants for a chromosome instability (CIN) phenotype. Proteasome subunit genes represented a major functional group, and subsequent analysis demonstrated an evolutionarily conserved role in CIN. Analysis of individual proteasome core and lid subunit mutations showed that the CIN phenotype at semi-permissive temperature is associated with failure of subunit localization to the nucleus. The resultant proteasome dysfunction affects chromosome stability by impairing the kinetics of double strand break (DSB) repair. We show that the DNA repair protein Mms22 is required for DSB repair, and recruited to chromatin in a ubiquitin-dependent manner as a result of DNA damage. Moreover, subsequent proteasome-mediated degradation of Mms22 is necessary and sufficient for cell cycle progression through the G_2_/M arrest induced by DNA damage. Our results demonstrate for the first time that a double strand break repair protein is a proteasome target, and thus link nuclear proteasomal activity and DSB repair.

## Author Summary

Chromosome Instability (CIN) is a genome phenotype that involves changes in chromosome number or structure, and accounts for most malignancies. In this paper, we describe a screen to identify a set of novel CIN genes and find that proteasomal subunits represent a major functional group. We show that proteasome dysfunction affects CIN by impairing DNA double strand break (DSB) repair. Previous studies speculated that the proteasome is required to degrade one or more components of the DSB repair machinery; however, until now, no such target has been identified. Here we identify the previously described CIN gene *MMS22* as a proteasomal target. We found that, as a result of DNA damage, Mms22 is ubiquitinated and recruited to chromatin. Mms22 then undergoes polyubiquitination and subsequent proteasome-mediated degradation. We also provide evidence that the degradation of Mms22 is important for the normal course of DNA repair and for exit from the G_2_/M arrest induced by DNA damage. Our results demonstrate for the first time that a DSB repair protein is a proteasome target, linking nuclear proteasomal activity and DSB repair. The mechanism of regulation of Mms22 may serve as a paradigm to understand how these additional proteins are regulated by the proteasome.

## Introduction

Genomic instability is recognized as being an important predisposing condition that contributes to the development of cancer [1]. A major class of genome instability is Chromosome Instability (CIN), a phenotype that involves changes in chromosome number and structure. Studies in yeast have shown that multiple overlapping pathways contribute to genomic stability [2]. The current view is that most spontaneous chromosomal rearrangements result from DSBs created mainly during DNA replication as a result of broken, stalled or collapsed replication forks [3]. In eukaryotes, DSBs are repaired either by Homologous Recombination (HR) or by Non-Homologous End Joining (NHEJ) mechanisms. Defects in either repair pathway result in high frequencies of genomic instability [4]. The HR pathway utilizes a homologous sequence to faithfully restore the DNA continuity at the DSB [5]. In contrast, NHEJ is a mechanism able to join DNA ends with no or minimal homology [6]. Recent studies suggest a role for the proteasome in DSB repair pathways: The Sem1/DSS1 protein is a newly identified subunit of the 19S proteasome in both yeast and human cells. In yeast, Sem1 is recruited to DSB sites with the 19S and 20S proteasome particles, and is required for efficient repair of DSBs by HR and NHEJ [7]. Human DSS1 physically binds to the breast cancer susceptibility protein BRCA2, that plays an integral role in the repair of DSBs, and is required for its stability and function and consequently for efficient formation of RAD51 nucleofilaments [8],[9].

The Ubiquitin-Proteasome System (UPS) is the supramolecular machinery that mediates the ubiquitin-mediated proteolysis of damaged or misfolded proteins, or of short-lived regulatory proteins. The 26S proteasome comprises the 20S core particle (CP) and the 19S regulatory particle (RP), which represent the base and lid substructures, respectively [10]. Nuclear targets that are degraded by the proteasome include proteins involved in pathways critical for chromosome integrity. For example, degradation of polyubiquitinated mitotic cyclin and of the anaphase inhibitor Pds1/securin allow sister chromatids to dissociate at the onset of anaphase [for a review see [11]]. The protein levels of the tumor suppressor protein p53 are also subtly controlled by ubiquitin-mediated degradation [12].

Previous studies suggest that the amino-terminal ubiquitin-like (Ubl) domain of Rad23 protein can recruit the proteasome for a stimulatory role during nucleotide excision repair (NER) in *S. cerevisiae*. It has also been shown that the 19S regulatory complex of the yeast proteasome can affect nucleotide excision repair independently of Rad23 protein [13]. Other studies suggested a model for the regulation of Xeroderma Pigmentosum protein C (XPC), which plays a role in the primary DNA damage sensing in mammalian global genome NER. According to this model the ubiquitin-proteasome pathway has a positive regulatory role for optimal NER in mammalian cells, and appears to act by facilitating the recruitment of XPC to DNA damage sites [13]–[18].

A putative role for the proteasome at DSB sites could be to degrade components of the DNA damage response after their function is completed. However, so far no protein involved in DSB repair has been described as a *direct* target of the proteasome.

In this paper, we describe a systematic screen of a recently released collection of temperature- sensitive (Ts) yeast alleles [19], to find a set of novel CIN genes. The screen and subsequent analysis of individual mutants revealed that proteasomal subunits represent a major functional group, with an evolutionarily conserved role in CIN. We found that the CIN phenotype is associated with a failure of proteasomes to localize to the nucleus in viable cells, and show that proteasome dysfunction affects chromosome stability by impairing the kinetics of DSB repair. We also identify the DNA repair protein Mms22 as a proteasome target, and demonstrate that the impaired DNA repair phenotype can be attributed to a failure in the recruitment and subsequent degradation of ubiquitinated chromatin-bound Mms22.

## Results

### CIN mutants from a new collection of Temperature sensitive (Ts) alleles in essential genes

**Figure 1:**
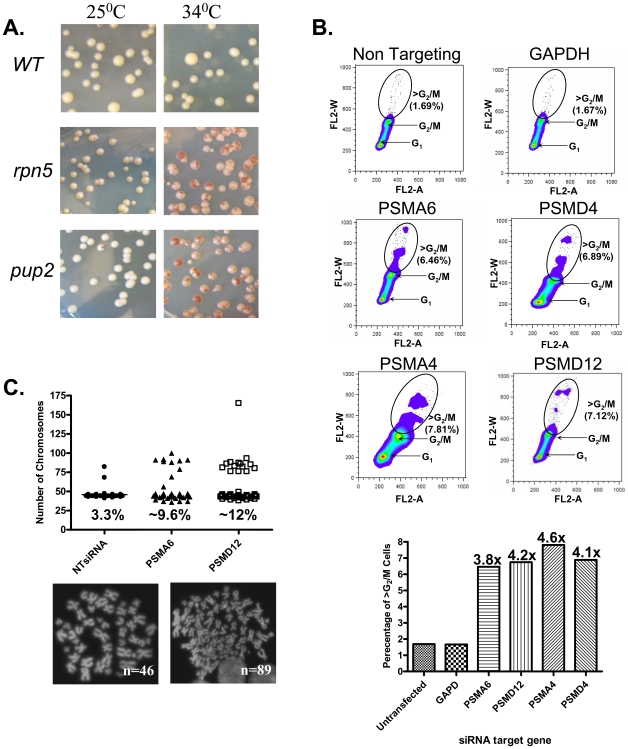
The CIN Phenotype of proteasome subunits is conserved from yeast to human cells. (A) Chromosome transmission fidelity (*ctf*) phenotype of yeast mutants defective for the proteasome subunits *pup2* and *rpn5ΔC* at a semi-permissive temperature (34°C) is scored by the appearance of sectored colonies, and compared to the isogenic wt strain. (B) DNA content dot plots of asynchronous HCT116 cells following siRNA knockdown in control, and test cases generated from cell populations harvested 5-days after transfection. HCT116 cell line, a mismatch repair-deficient cell line, was used, as it is a chromosomally stable, near diploid colorectal cell line that does not inherently exhibit CIN. Cells were labeled with propidium iodide and subjected to flow cytometry. Circles delineate the population of cells having >G_2_/M DNA contents. The graph summarizes the relative increase in this cell population as compared to the non-targeting and *GAPDH* controls. (C) Scatter plot depicting the total chromosome number distribution after targeted knockdown of *PSMA6*, *PSMD12*, or a non targeting (NT) RNAi control. Percentage of mitotic spreads with greater than 46 chromosomes is indicated at the base of each column; (below) Representative images of DAPI-stained mitotic spreads from untransfected cells (N = 46 chromosomes) and aneuploid cells after treatment with PSMA6 siRNA (N = 89 chromosomes).

In this study we expanded a recent screen for mutants affecting chromosome stability [19], by assessing the chromosome transmission fidelity (Ctf) phenotype (for details see Materials and Methods) in an additional 208 Ts strains. The functional distribution of the identified genes reveals that proteasome subunits are highly represented (Figure 1A and Table S1), we therefore decided to examine the mechanisms by which mutations in proteasome subunits cause CIN.

### Diminished levels of proteasome subunits in mammalin cells causes CIN

To test whether the CIN phenotype associated with proteasome dysfunction is evolutionarily conserved, we examined whether diminished proteasome subunit levels would cause a CIN phenotype in human cell lines. Small interfering RNAs (siRNAi) were used to target two human proteasome core (*PSMA6* and *PSMA4*) and two lid subunits (*PSMD4* and *PSMD12*) in the HCT116 cell line. To reduce the off-target effect, each experiment was performed with the two most effective siRNA duplexes (pointed by black arrows in Figure S1A). As shown in Figure 1B, relative to the controls, knockdown of Psma6, Psma4, Psmd4 and Psmd12 resulted in an increase in the frequency of cells with DNA contents greater than that of G_2_/M cells. Chromosome spreads after targeted knockdown of *PSMA6* and *PSMD12* established that the increase in DNA content is due to a dramatic increase in the number of cells with a total chromosome number above 46 (Figure 1C). Taken together, these results suggest that the proteasome lid and core components have a role in chromosome stability maintenance.

### Proteasome CIN mutations cause nuclear mislocalization

**Figure 2:**
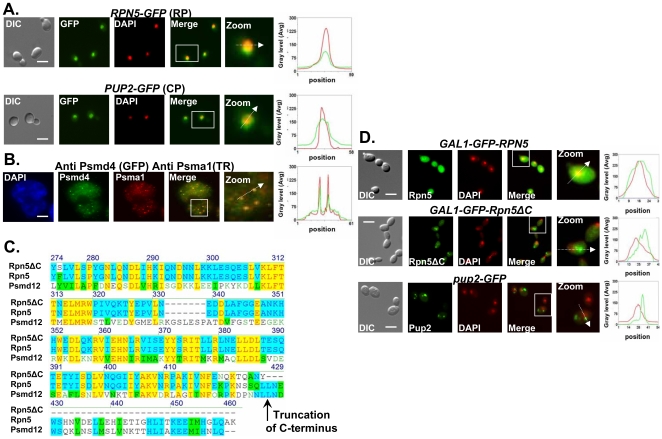
Proteasome subunits in yeast and mammalian cells localize to the nucleus; the Ts allele of *rpn5* is truncated at the C-terminus; proteasome CIN mutants show nucleus mislocalization of proteasome subunits. (A,B) Proteasome subunits in yeast and mammalian cells localize to the nucleus. (C) The Ts allele of *rpn5* is truncated at the C-terminus. (D) Proteasome CIN mutants show nucleus mislocalization of proteasome subunits. The panels represent high resolution (x100) representative images of yeast or mammalian cells. A region identified by the white box is further magnified (zoom panel). The position of the white arrow within the zoom panel delineates the line scan that was used to quantitate the fluorescent signal intensities per pixel in the line scan graphs (right panel). Unless otherwise stated, all the images represent a 3-D projection of x100 Z-series images extending above and below the entire nucleus. Scale bars, 3 µm. (A) Logarithmic yeast cultures were permeabilized and DNA was DAPI stained to mark the nucleus. The panels depict the nuclear localization of the yeast Regulatory Particle (RP) Rpn5-GFP, and Core Particle (CP) Pup2-GFP. GFP and DAPI are represented by green and red curves in the line scan graphs, respectively. (B) Nuclear enrichment and foci colocalization of immunofluorescently labeled mammalian proteasomal subunits Psma1 (CP) and Psmd4 (RP). Cells were DAPI stained and visualized by GFP, Texas Red (TR) and DAPI. Red and green lines represent TR and GFP, respectively. Panels represent a 3-D projection of x100 Z-series images extending above and below the entire nucleus. (C) The *rpn5-*Ts allele was sequenced and its predicted translation product aligned to the wt yeast protein Rpn5, and its human homolog, Psmd12. The truncation point of Rpn5ΔC is indicated by a black arrow. (D) Localization analysis of N-terminal GFP fusion of Rpn5ΔC, and Rpn5 control (both expressed from a galactose-inducible promoter). The images depict the localization of GAL1-GFP-Rpn5 *vs.* GAL1-GFP-Rpn5ΔC. GFP-Rpn5 localizes to the nucleus (overlap between the DAPI and GFP channels). Lack of overlap in GFP-Rpn5ΔC indicates nuclear mislocalization. Similar results were obtained for *pup2*-Ts (Pup2-GFP) (compare to Figure 2A).

Previously it was established that the 26S proteasome localizes to the nucleus [20]. Here we confirmed the nuclear localization of the proteasomal lid and core subunits both in yeast and human cells (Figure 2A and 2B). The CIN phenotype caused by Ts alleles of proteasome subunits suggests that a nuclear function of the proteasome is impaired in the mutants. Sequence analysis of the *rpn5* Ts allele reveals a single base pair insertion that introduces a premature stop codon, resulting in truncation of 39 amino acids at the C-terminus (Figure 2C). To analyze the localization of this truncated form, termed *rpn5ΔC*, GFP was fused in frame at its N-terminus. As a control, an identical N-terminal GFP fusion was constructed for the wt *RPN5* gene (both expressed from a galactose-inducible promoter). The results show that whereas the control GFP-Rpn5 protein localizes predominantly to the nucleus, GFP-Rpn5ΔC localizes predominantly to the cytoplasm (Figure 2D). Similar nuclear mislocalization results were obtained for the mutated core subunit, Pup2Ts-GFP (Figure 2D). The mislocalization of the *rpn5ΔC* mutant protein indicates that the C-terminal domain (CTD) is important for Rpn5 nuclear localization in yeast.

### Mutated proteasome subunits affect DNA DSBs repair kinetics

**Figure 3:**
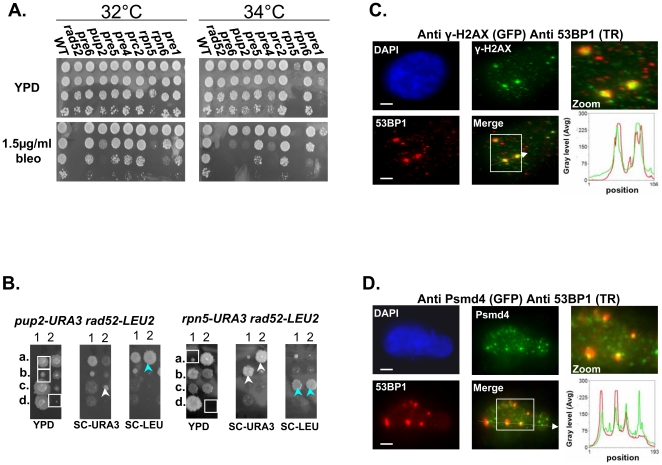
Mutated proteasome subunits affect the repair of DNA DSBs. (A) Most of proteasomal Ts mutants are sensitive to bleomycin (bleo). Five-fold serial dilutions of the indicated proteasomal subunits mutants were spotted on YPD medium lacking or supplemented with 1.5 µ/ml of bleo. Cells were incubated at 32°C and 34°C to find the semi-permissive temperature of each Ts mutant. (B) *rpn5ΔC* and *pup2* show synthetic growth defect with *rad52*. To examine whether there could be a link between the proteasome and the repair of DSBs, we created and sporulated heterozygous diploid strains containing Ts alleles of either *rpn5ΔC* and *pup2* combined with *rad52*. Tetrad dissection showed that Ts alleles of *rpn5ΔC* and *pup2* cause a synthetic growth defect when either one is combined with *rad52*. The synthetic growth defect of the double mutant spores (encircled by white squares on the YPD plate) is evident when compared to the single haploid mutants (pointed out by white or light blue arrows). (C,D) Protesomal subunits associate with DSB markers in mammalian cells. HeLa cells were treated for 2 hrs with 5 µ/ml of bleo prior to subjection to IIF microscopy. Primary antibodies were recognized with appropriate secondary antibodies conjugated with either Alexa-fluor 488 (GFP filter), or Cy-3 (TR filter). Scale bars, 3 µm. (C) IIF to demonstrate the colocalization pattern of 53BP1 and γ-H2AX in bleo-treated cells. The DSB markers 53BP1 and γ-H2AX show clear co-localization at large foci, likely to represent DSB sites. Red and green curves on the line scan graph represent 53BP1, and γ-H2AX respectively. (D) Representative images demonstrating an association of the RP subunit Psmd4 with DSB sites, represented by the large 53BP1 foci. Red and green curves on the line scan graph represent 53BP1 and Psmd4 respectively.

Next we wanted to address the underlying defect in proteasome function that results in CIN. First we examined the proteasomal CIN mutants for sensitivity to Bleomycin (bleo) [21], and to hydroxyurea (HU)[22]. Mutants involved in DSB repair are usually sensitive to both drugs [23],[24]. We show that at semi-restrictive temperatures all proteasome mutants display varying degrees of sensitivity to these drugs (Figure 3A and Figure S1B). These results support a previous study showing that other proteasome mutants show sensitivity to DNA damaging agents [7]. Moreover, Ts alleles of *rpn5ΔC* and *pup2*, display a synthetic growth defect when either one is combined with *rad52*, a key factor in the DSB repair pathway [25] (for details see Figure 3B).

In support of a role for the yeast proteasome in DSB repair, previous ChIP experiments have provided evidence for the recruitment of the proteasome to DSB sites [7]. To test whether this phenomenon is conserved in mammalian cells, we performed Indirect ImmunoFluorescent (IIF) on Hela cells treated with Bleo, to look at the association of the RP subunit Psmd4 with DSB sites, represented by 53BP1 large foci (Figure 3D). In 183/200 53BP1 large foci counted, the Psmd4 focus was peripherally associated with the DSB site (Figure 3D). As a control we analyzed a similar number of unchallenged cells; in this case a significantly lower number of 53BP1 foci (44) could be detected, 21 of which were associated with a Psmd4 focus. As was previously shown [26],[27], these foci likely represent spontaneous DNA DSBs generated during DNA replication. In addition, we quantitated the signals of 50 Psmd4 foci that were associated with 53BP1 as a result of Bleo treatment; in 95% of the cases this signal was 5–10 times more intense than the average signal representing the Psmd4 foci not associated with 53BP1. These results provide evidence for an association of the proteasome with DSBs sites in human cells.

**Figure 4:**
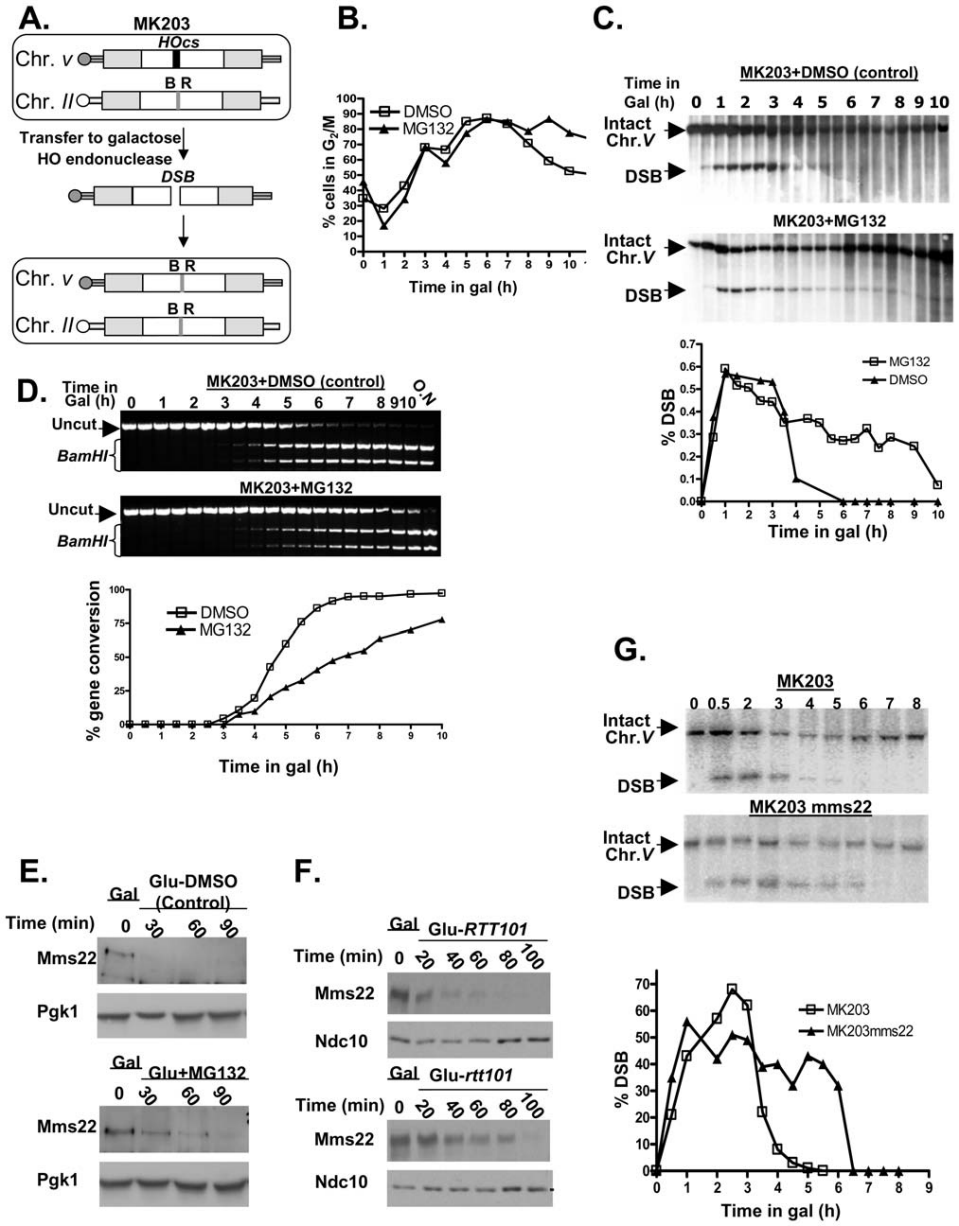
Proteasome mutants exhibit defective DSB repair kinetics; the turn over of Mms22 is regulated by the proteasome; Mms22 plays a role in DSB repair. (A–D) Proteasome mutants exhibit defective DSB repair kinetics. (E,F) The turn over of Mms22 is regulated by the proteasome. (G) Mms22 plays a role in DSB repair. (A) Schematic representation of MK203 (for more details see Materials and Methods, DSB repair kinetic experiments). White rectangles represent the *ura3* alleles on chromosomes *II* and *V*. A black bar within the *ura3* alleles represents the HO cut site (HOcs); a grey bar depicts the inactive HOcs-inc flanked by the *BamHI* (B) and *EcoRI* (R) restriction sites. Transfer of the cells to galactose-containing medium results in a DSB that is repaired by homologous recombination. (B–D) DSB repair kinetics of MK203 cells in the presence of proteasome inhibitor. MK203 *pdr5* cells were grown to mid-logarithmic phase in glycerol-containing medium (gly) (no HO-induction) containing 20 mM MG132, or DMSO control. Cells were then transferred to galactose-containing medium (gal; constitutive HO-induction and DSB formation at the *URA3* locus) containing the same concentration of the drug. Samples were collected for analysis at timely intervals, and subjected to microscopic examination and Southern blot analysis. (B) Microscopic examination of dumbbell shaped cells indicates the percentage of G_2_/M in the control, or cells subjected to MG132, at each of the indicated time points. (C) Southern blot analysis and quantification graph (bottom) of the DSB repair kinetics in MK203 cells treated with MG132. (D) PCR analysis of the kinetics of the gene conversion product formation. PCR reaction followed by *BamHI* restriction digest detect the final step of the repair, which is the re-ligation of the broken ends and transfer of the two polymorphic restriction sites on either side of the HOcs from chromosome *II* to chromosome *V* [29]. MG132 treated cells show a delay in gene conversion product formation, as apparent from the quantification graph (bottom). (E) Western Blot detects the levels of Mms22 following a *GAL1* promoter shut-off chase experiment. The expression of *GAL1*-HA-Mms22 was induced by growing the cells in 2% galactose (Gal) for 3 hours (t-0). Cells were released into 2% glucose to shut-off the expression of Mms22. Glucose was supplemented with 20 mM MG132, or with DMSO (control), Pgk1 was used as a loading control. (F) *RTT101* regulates the levels of Mms22. *GAL1* shut-off chase experiment was performed as in (E), this time wt cells *vs. rtt101* strains were released into 2% glucose. Ndc10 was used as a loading control. (G) Southern blot analysis and quantification graph (bottom) of the DSB repair kinetics in wt MK203 cells versus *mms22*.

In order to examine the nature of the observed difference in DSB repair under proteasome dysfunction we studied the effect of the well-characterized proteasome inhibitor, MG132 [28] on the repair kinetics of a single defined chromosomal break in the yeast genome using the strain MK203 [29] (for more details see Figure 4A, and Materials and Methods). The strain used carried a mutation in the *PDR5* gene, to prevent the cells from pumping the drug out of the cell [30].

As previously described [29], the control and MG132-treated cells arrest at G_2_/M three hrs after DSB induction. However, while the control cells exited from the arrest after 8 hrs, MG132-treated cells remained arrested even 10 hrs after DSB induction (Figure 4B). Southern blot analysis detected complete repair of the broken chromosome by 5 hrs following induction in control cells. In contrast, MG132-treated cells exhibited only partial repair of the DSB. Nine hours after transfer to galactose (which induces DSB repair), more than 30% of the cells still carry a broken chromosome (Figure 4C). At later times this proportion is reduced, probably due to outgrowth of cells with a repaired chromosome *V*.

We next examined the kinetics of formation of the gene conversion (GC) repair product (Figure 4D). In the control cells, GC can be detected 3.5 hrs after DSB induction, and the whole cell population was completely repaired by 6.5 hrs. In contrast, in MG132-treated cells only 70% of the cells exhibited repair 10 hrs after DSB induction (Figure 4D). Taken together, these results demonstrate that inhibition of proteasome activity affects the ability of yeast cells to carry out repair of a DSB, resulting in a prolonged cell cycle arrest. Moreover, MG132-treated cells also exhibit a higher level of CIN, measured using the a-faker-like (ALF) genome instability test [31] (Figure 2SA).

### The expression of Mms22 is regulated by the Ubiquitin-Proteasome System (UPS)

One possible explanation for the requirement of an active proteasome to complete the DSB repair is that the proteasome could be required to degrade one or more components of the DSB repair machinery. We looked for potential proteasome targets with a role in DSB repair. Such a target is expected to exhibit phenotypes that include both CIN (similar to that of proteasomal mutants), and sensitivity to DNA damaging agents, such as ionizing radiation or radiomimetic drugs such as methyl methanesulfonate (MMS). We recently used the *Sacharomyces cerevisiae* deletion collection to systematically screen for mutants exhibiting a CIN phenotype [31]. The *mms22* mutant, which shows sensitivity to several DNA damaging agents that cause DSBs [32],[33] was among the mutants exhibiting the strongest CIN phenotype. To test whether Mms22 is a substrate of the proteasome, a strain carrying an inducible tagged protein (*GAL1*-HA-Mms22) was subjected to a promoter shutoff experiment. Figure 4E shows that under these conditions in wt cells Mms22p is degraded; in contrast, in the presence of MG132, the level of Mms22 protein stays high, and appears to be degraded to a lesser degree.

To further assess *MMS22* function, we conducted a two-hybrid screen using Mms22 as the bait. This approach identified Rtt101/Cul8 as a protein that interacts with Mms22. We confirmed this interaction by IP. Consistent with a recent study [34], we concluded that Mms22 and Rtt101 proteins interact *in vivo* (Figure S2B and S2C). Rtt101 is one of four cullins in *S. cerevisiae*, with demonstrable ubiquitin ligase activity *in vitro*, but as yet no known substrate *in vivo* [35]. Based on the physical interactions seen between Mms22p and Rtt101, it has been suggested that Mms22 is a functional subunit of the Rtt101-based ubiquitin ligase [34]. Our results show that Mms22 is targeted by the proteasome; we therefore hypothesized that the turnover of Mms22 could be mediated by the Rtt101 E3 ubiquitin ligase complex. A promoter shut-off chase was used again to analyze the stability of the Mms22 protein in the presence or absence of the Rtt101 cullin. Figure 4F shows that Mms22p accumulated to a higher level during the induction period in *rtt101* mutants in comparison to wt cells. To rule out the possibility that only the overexpressed proteins were being degraded by the proteasome, and to show that similar results can be observed in the context of endogenous levels of Mms22, we have performed cyclohexamide chase experiments in cells expressing Mms22-HA (Figure S2D). Western blot analysis revealed that, as observed in the *GAL*-driven overexpression experiments, Mms22 is also degraded in wt cells. Notably, Mms22 accumulated to higher levels in the presence of MG132, or in a *Δrtt101* background. These results clearly demonstrate that the turnover of Mms22 is regulated by the Ubiquitin-Proteasome System (UPS), and mediated by the Rtt101 cullin.

### Mms22 plays a role in DSB repair

*mms22* cells show sensitivity to several DNA damaging agents that cause DSBs [32],[33]. To directly examine the kinetics of DSB repair in *mms22* mutants, we used the MK203 system again, to compare repair kinetics of wt *vs. mms22* cells following induction of a DSB. As seen in cells under proteasome inhibition, *mms22* mutants show a delay in the disappearance of the broken chromosome compared to wt cells (Figure 4G). Additionally, and also similarly to MG132-treated cells, *mms22* cells show a difference in gene conversion kinetics compared to wt cells (Figure S2E).

### Ubiquitination of Mms22 is induced by DNA damage in a *RTT101* dependent manner

**Figure 5:**
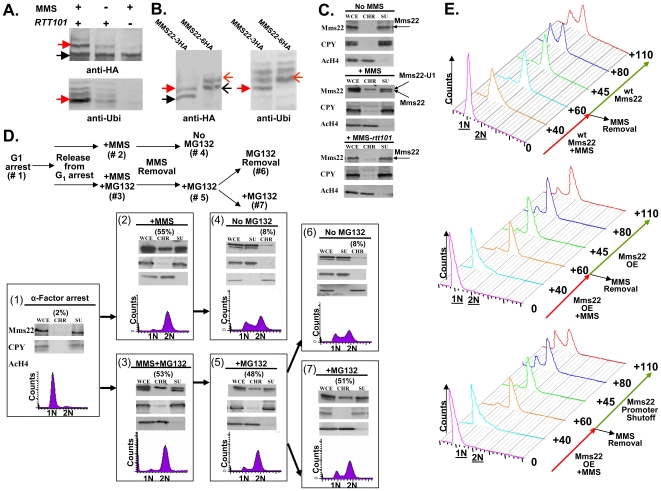
Ubiquitination of Mms22 is induced by DNA damage in a *RTT101* dependent manner; Mms22 is recruited to chromatin upon DNA damage in a *RTT101* dependent manner; degradation of Mms22 from chromatin is associated with exit from the DNA damage induced G_2_/M arrest. (A, B) Ubiquitination of Mms22 is induced by DNA damage in a *RTT101* dependent manner. (C) Mms22 is recruited to chromatin upon DNA damage in a *RTT101* dependent manner. (D,E) Degradation of Mms22 from chromatin is associated with exit from the DNA damage induced G_2_/M arrest. (A) 3HA*-*tagged Mms22 cells were grown in the presence of 20mM MG132, with or without 0.025% MMS, and subjected to IP. After electrophoresis on a low percentage gel (6%), the precipitated protein was blotted to a membrane which was successively immunobloted with an anti-HA, and an anti-Ubi antibody. Black arrow labels Mms22-3HA, red arrow labels the modified form of Mms22-3HA. (B) *In vivo* demonstration that the ubiquitinated proteins observed in (A). are a series of polyubiquitinated forms of Mms22. Cells carrying Mms22 tagged with either 3HA, or 6HA were grown in the presence of 20 mM MG132, and 0.025% MMS, and subjected to IP. After electrophoresis the precipitated proteins were blotted to membranes which were subjected to immunoblotting with anti-HA, and anti-Ubi antibodies. Black and open black arrows label Mms22-3HA and Mms22-6HA respectively. Red and open red arrows label the mono-ubiquitinated form of Mms22-3HA and Mms22-6HA respectively. (C) Cell extracts (WCE) were separated into supernatant (SU) and chromatin (CH) fractions. HA-tagged Mms22 was detected by immunoblotting in wt (middle), or *rtt101* deleted cells (right), treated with 0.025% MMS, and compared to the untreated wt control (left). Anti Carboxy peptidase-Y (CPY), and Anti Acetylated Histon H4 (AcH4) served as a SU and CH fractions controls respectively. (D) (Top)-Experimental design. Chromatin fractionation assay was performed as in (B). The quantity of Mms22 on the chromatin bound fraction is represented as percentage of the WCE. Cells were synchronized to G_1_ (#1), and released from the arrest in the presence of 0.025% MMS (#2), or MMS+MG132 (#3). Next, MMS was removed, and samples were allowed to recover in the presence (#5), or absence (#6) of MG132. Sample #6 and #7 represent a division of sample #6 to a sample without, or with MG132 respectively. (E) Failure to degrade Mms22 impairs progression of repair, leading to prolonged cell cycle arrest. G_1_ arrested cells were released into YEP-Gal medium (inducing overexpression of *GAL1-MMS22* cells) containing 0.025% MMS. MMS was washed from the G_2_/M arrested cells, and cells were allowed to recover in YEP-Gal or YEP-Glu (thus keeping either high or low expression levels of Mms22 in *GAL1-MMS22* cells respectively). Top panel: wt expression levels of Mms22. Middle panel: Mms22 was over-expressed (OE) before, and after the removal of MMS. Bottom panel: Mms22 was OE before the removal of MMS, while its *GAL1* promoter was shut off following MMS removal. Samples were collected at timely intervals and subjected to FACs analysis; numbers along the red and green arrows represent the time (minutes) since the release from the G_1_ arrest, or following MMS removal respectively.

Substrates are usually targeted for degradation by the proteasome by polyubiquitination [10]. To test the ubiquitination levels of Mms22, we performed the experiments described in Figure 5A and Figure S2F. IP of Mms22-HA followed by immunoblotting reveals an additional band which migrates slower than Mms22-HA. This band most probably represents the ubiquitinated form of Mms22, as revealed by successive immunoblotting with an anti-Ubi antibody (Figure 5A). Additional proof that this band represents ubiquitinated Mms22 was obtained by successive immunoblotting with an anti-Myc antibody in a strain carrying a myc-tagged version of the ubiquitin protein (Figure 2SF). The results also show that Mms22 ubiquitination is *RTT101* dependent. Finally, a 4 -fold increase in Mms22 ubiquitination was observed when cells were exposed to DNA damage, suggesting that the ubiquitination of Mms22 plays a functional role in DNA repair (Figure 5A).

To provide a rigorous *in vivo* demonstration that the ubiquitinated proteins observed by Western blotting were indeed a series of polyubiquitinated forms of Mms22, we performed the following experiment in which the 3HA and 6HA tagged versions of Mms22 were used in parallel. Cells were grown in the presence of MMS, and subjected to IP followed by immunoblotting with anti-HA. As expected from the results shown in Figure 5A, treatment with MMS led to an additional band which migrated more slowly than the band representing Mms22. Importantly, this band changed its electrophoretic mobility upon switching the tag on Mms22 from 3HA to 6HA, demonstrating unequivocally that it represents a specific *in vivo* modification of Mms22 (Figure 5B, left). This modification is indeed the specific ubiquitination of Mms22 as revealed by a similar electrophoretic shift of the bands that appeared following a successive immunoblotting with an anti-Ubiquitin antibody (Figure 5B, right). Alignment of the anti-HA and anti-Ubi antibody membranes, and the observed co-alignment of the electrophoretic shifts characteristic of the differentially tagged Mms22 protein species, indicates that the additional band that migrates slower than Mms22-HA is the mono-ubiquitinated form of Mms22.

### Upon exposure to DNA damage Mms22 is associated with chromatin in a *RTT101-* dependent manner

Genome-wide genetic interaction results have shown that *MMS22* clusters with *RTT109*, and *ASF1* [36], two proteins required for histone H3 modification [37]. We therefore hypothesized that the ubiquitinatation of Mms22 may facilitate its recruitment to chromatin upon DNA damage. To test this idea we separated whole cell extracts (WCE) into soluble (SU) and chromatin-bound (CHR) fractions. Fractions were then subjected to immunoblotting using anti HA (Mms22-HA). The results clearly show that in unchallenged cells Mms22 is mainly present at the SU fraction (Figure 5C top). Treatment with MMS, however, leads to an enrichment of ubiquitinated Mms22 on the chromatin-bound fraction (Figure 5C middle). This enrichment was significantly reduced in the absence of *RTT101* or *RTT109* (Figure 5C bottom and Figure S3A).

Taken together, our results suggest that the ubiquitinated form of Mms22 on chromatin plays a functional role in dealing with DNA damage. A similar experimental approach was used to show chromatin enrichment of the proteasomal lid subunit Rpn5 upon exposure to MMS (Figure 3SB), which is consistent with ChIP analysis of proteasomal subunits at induced DSBs sites [7].

### Mms22 degradation by the proteasome is important for its function in DNA repair

DNA damage induces the recruitment of both Mms22 and the Proteasome to chromatin, predicting that Mms22 degradation by the proteasome plays an important role in performing DNA repair. To test this idea we performed the experiment described in Figure 5D. MMS treatment resulted in G_2_/M arrest, and a chromatin fractionation assay revealed that Mms22 was recruited to chromatin in the presence, or in the absence, of MG132 (Figure 5D-2 and 5D-3). These results indicate that the recruitment of Mms22 to chromatin is proteasome-*independent*. The recruitment to chromatin is not cell cycle dependent, since a similar recruitment of Mms22 to chromatin was detected even when cells were kept in G_1_ during MMS treatment (data not shown). In contrast, the exit from the G_2_/M arrest following the removal of MMS was proteasome *dependent*, since only removal of MG132 from the medium led to the degradation of Mms22 from chromatin, which was associated with the exit from the G_2_/M arrest (compare 6D-5 *vs.* 6D-6 and 6D-7). To rule out the possibility that the prolonged exposure to MG132 and not MMS treatment led to the G_2_/M accumulation, we performed the control experiment described in Figure S3C. We show that samples released from the G_1_ arrest and constantly exposed to MG132 continued cycling normally in contrast to samples from a similar time point exposed to MMS+MG132 (compare Figure 5D-5 to Figure S3C).

Next we wanted to test whether the correlation between the accumulation of Mms22 on chromatin, and the failure to recover from cell cycle arrest upon DNA damage can be attributed (among other factors) to the specific accumulation of Mms22 in cells with defective proteasome activity. We therefore tested whether overexpression (OE) of Mms22 (which simulates the accumulation of Mms22 in proteasome mutants) also results in impaired recovery from DNA damage induced by MMS. While wt cells start to recover from the G_2_/M arrest 80 min after the removal of MMS from the medium (Figure 5E top), cells that overexpress Mms22 were still arrested even after 110 min (Figure 5E middle). Importantly, the removal of MMS together with Mms22 promoter shutoff (leading to the degradation of Mms22, data not shown), led to enhanced recovery from the G_2_/M arrest, when compared to cells still overexpressing Mms22 (Figure 5E middle versus bottom).

**Figure 6:**
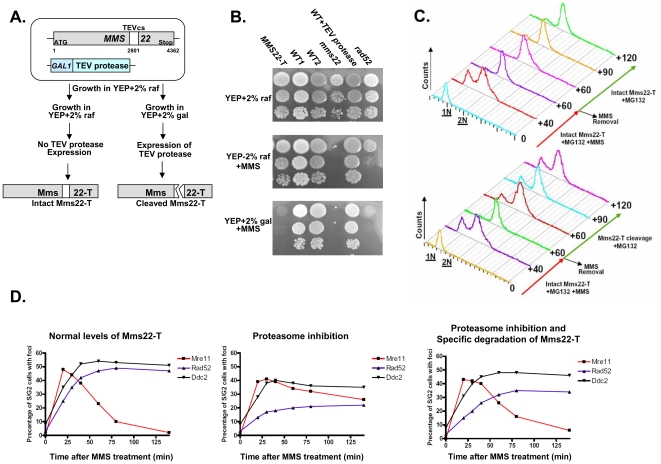
Degradation of Mms22 is sufficient to allow exit from the DNA damage induced G_2_/M arrest. (A,B) Schematic representation of the experimental design, and growth phenotypes. The TEV protease consensus cleavage site (cs) was introduced at position 2801 of Mms22 which is expressed from its endogenous promoter (*MMS22*-T). The cells also contain the TEV protease under the control of the inducible *GAL* promoter [39]. YEP medium supplemented with 2% raffinose (raf) suppresses the expression of the TEV protease, and keeps Mms22-T functional, as indicated by its normal growth on YEP+raf+MMS media (compare to *mms22* strain). Transfer of the cells to medium containing 2% galactose (gal) results in TEV protease induction, and the specific cleavage of Mms22-T. The inactivation of Mms22-T is indicated by its impaired growth on YEP+gal+MMS medium. (C) G_1_ arrested cells were released into YEP-raf medium (which blocks the expression of the TEV-protease), containing 0.025% MMS and 20 mM MG132. MMS was then washed from the G_2_/M arrested cells, and cells were allowed to recover in YEP-raf (top), or YEP-gal (bottom) (intact, or specific cleavage of Mms22-T respectively), both supplemented with MG132. Samples were collected at timely intervals and subjected to FACS analysis. Numbers along the red and green arrows represent the time (minuets) since the release from the G_1_ arrest, or following the removal of MMS respectively. (D) Temporal analysis of Mre11, Ddc2, and Rad52 focus formation following DNA damage and proteasome inhibition. Yeast strains containing Mms22-T, *GAL* inducible TEV-protease, and a YFP tagged version of either Mre11, Ddc2, or Rad52 were released into non-inducing YEP-raf medium containing 0.025% MMS and 20 mM MG132 (intact Mms22-T). Following the induction of DNA damage MMS was washed from the media, and cells were allowed to recover in YEP-raf (left: wt levels of Mms22), YEP-raf+MG132 (middle: accumulation of intact Mms22-T), or YEP-gal+MG132 (right: inducing the specific cleavage of Mms22-T by the TEV protease). Following the removal of MMS, samples were collected at timely intervals, fixed, and subjected to fluorescent microscopy. At each of the indicated time points at least 150 S/G_2_ cells (budded) were analyzed for the presence/absence of Mre11, Ddc2, or Rad52 foci.

Having shown that degradation of Mms22 can promote exit from the G_2_/M arrest, we tested whether the specific degradation of Mms22 is sufficient for the exit. We created yeast strains carrying an allele of Mms22 (Mms22-T) that is expressed from its endogenous promoter and can be cleaved by the tobacco etch virus (TEV) protease [38],[39]. This protease can be conditionally expressed (for details see Figure 6A). Induction of the TEV protease leads to cleavage and inactivation of the Mms22-T protein (Figure 6A and 6B). When we conditionally expressed the protease in the presence of MG132 in cells arrested in G_2_/M as a result of MMS treatment, the cleavage of Mms22-T resulted in a clear release from the DNA damage-induced G_2_/M arrest (Figure 6C, compare top and bottom panels). Thus, degradation of Mms22 is essential for release from the cell cycle arrest induced by DNA damage.

### Mms22 degradation by the proteasome is essential for the progression of DNA repair

We have shown that the degradation of Mms22 is essential for release from damage-induced cell cycle arrest. Next, we tested whether the prolonged G_2_/M arrest is also associated with impaired response to DNA damage. Using yeast strains tagged with fluorescent versions of three major components of the DNA DSB repair machinery: Mre11, Ddc2, Rad52, we conducted temporal analysis of focus formation following DNA damage (Figure 6D). Consistent with previous data [40] we show that Mre11 (a member of the MRX complex) is the earliest protein to form foci. Mre11 foci formation is followed by later recruitment of Ddc2 (the yeast orthologue of human ATR-interacting protein ATRIP) and the repair protein Rad52. We show (Figure 6D, left) that in wt cells, as Rad52 and Ddc2 were recruited, Mre11 foci disassembled. This disassembly was circumvented when cells were exposed to a proteasome inhibitor, and led to delayed and reduced focus formation of Rad52 (Figure 6D, middle). Remarkably, the *specific* degradation of Mms22 resulted in a clear disassembly of Mre11 foci and recovery of Rad52 foci (Figure 6D, right). Our results demonstrate that degradation of Mms22 is essential for the normal course of DNA DSB repair, and for the release from the cell cycle arrest induced by DNA damage.

## Discussion

We describe the first systematic screen of a recently released resource (still under development) consisting of Ts mutants of all essential yeast genes for which no Ts-allele had previously been isolated [19]. Among the 40 genes identified, 8 encoded proteasomal subunits. Genetic and biochemical analysis showed that CIN was associated with the failure of proteosomal subunits to localize to the nucleus, impaired kinetics of DSB repair, and failure to turnover the DNA repair protein Mms22 targeted for degradation by the proteasome.

Recent studies have suggested a role for the proteasome in the repair of DSB in yeast [7], and mammalian cells [41],[42]. In our current work, we show that mutations in the proteasome subunits *rpn5ΔC* and *pup2*, which cause nuclear mislocalization, are associated with impaired DSB repair. All other proteasomal Ts mutants tested were sensitive to drugs inducing DSBs, implying that the proteolytic activity of the proteasome is required for DNA repair. By examining the kinetics of DSB repair in cells treated with the proteasome inhibitor MG132, we obtained evidence for delayed kinetics of repair (Figure 4C). We showed that both the disappearance of the break as well as the kinetics of formation of the gene conversion product were delayed in treated cells compared to untreated cells (Figure 4D). As MG132-treated cells arrest in G_2_/M similarly to untreated cells, it is evident that checkpoint regulation due to DSB is not impaired in the treated cells. The delay in DSB repair suggests that proteasome activity might be required for the regulation of the DNA repair machinery.

A potential role for regulation of DSB repair by the proteasome in mammalian cells is supported by a recent study showing that proteasome inhibition affected the choice of HR repair pathways [41]. A different study showed that proteasome-dependent protein degradation substantially contributes to HR but not NHEJ [42]. It is tempting to speculate that the proteasome accumulates at sites of DSB, and that its proteolytic activity is required to degrade one or more components of the DSB repair machinery, or DNA damage response/repair proteins.

To date no protein involved in DSB repair has previously been described as a *direct* target of the proteasome. In this study, we identify Mms22, a protein required for efficient repair of DSBs (Figure 4G and Figure S2E), as a direct target of the proteasome degradation pathway (Figure 4E and 4F and Figure 2SD). Recently, Zaidi and colleagues [34] showed that Mms22 physically interacts with Rtt101, and suggested that Mms22 is a functional component of the SCF^rtt101^ ligase, perhaps as a substrate specificity factor. Although our studies do not address whether Mms22 is a subunit of SCF^rtt101^, we show clear evidence that Mms22 is a substrate of the SCF^rtt101^ and that proteasome-mediated turnover of Mms22 is important for the process of DNA repair.

The effect of *MMS22* accumulation on the course of DSB repair (Figure 6D) suggests that Mms22 activity facilitates the recruitment of the HR machinery to DSBs. Histone modification occurs readily at sites of DSB or UV damage [43],[44] and it is becoming increasingly clear that proper chromatin handling is essential for successful repair. Indeed, we show that DNA damage results in Mms22 recruitment to the chromatin bound fraction (Figure 5C). Importantly, our results also show that recruitment of Mms22 to chromatin is not sufficient for the normal course of DNA repair, and that an essential step is a proteasome-mediated degradation of Mms22. These results thus identify for the first time a proteasome target that links proteasomal nuclear activity and DNA double strand break repair.

We propose the following model for the mechanism by which nuclear activity of the proteasome contributes to repair of DSBs. DNA damage results in a SCF^rtt101^ E3 ubiquitin ligase-dependent accumulation of the ubiquitinated form of Mms22 on chromatin that, as suggested above, plays a role in dealing with DNA damage. Subsequent degradation of ubiquitinated Mms22 by the proteasome is an important step in completion of the DNA repair process. Once Mms22 executes its function in DNA repair it becomes a target for degradation by the UPS, and is removed from chromatin. Failure to degrade Mms22 results in impaired DNA repair and prolonged cell cycle arrest. In support of our model, we show that reactivation of an inhibited proteasome results in degradation of the accumulated chromatin-bound Mms22, and in recovery from the G_2_ arrest induced by DNA damage (Figure 5D).

The synthetic genetic interaction that we describe for the proteasome and the *rad52* mutant points to additional roles of the proteasome in DNA repair. Given its central role in protein degradation, it is indeed very likely that, in addition to Mms22, the proteasome regulates additional proteins involved in DNA repair. In this regard, proteasome inhibition in combination with DNA damage probably results in the accumulation of many proteins besides Mms22, which altogether may lead to the impaired recovery from the cell cycle arrest. We show, however, that specific accumulation of Mms22 by overexpression causes defects in recovery from DNA damage-induced G_2_/M arrest, whereas turnover of Mms22 after promoter shutoff allows recovery to occur (Figure 5E). Moreover, we also show that the specific degradation of Mms22 in the presence of proteasome inhibitor is sufficient for the exit from the DNA damage-induced G_2_/M arrest (Figure 6C). The arrest by itself was not affected by proteasome inhibition, which is consistent with the normal kinetics of foci formation of the checkpoint protein Ddc2. In contrast, proteasome inhibition affected the disassembly of Mre11, which in turn impaired the recruitment of the repair machinery, as demonstrated by the kinetics of Rad52 foci formation (Figure 6D). A similar phenotype was previously reported for *Δsae2* mutants, supporting the notion that Sae2 is required for the transition from Mre11 binding to the recombinational repair function carried out by Rad52 [40],[45]. Our results suggest that degradation of Mms22 occurs at the same transition stage and that when this transition is impaired, cells can no longer proceed with the normal course of DNA repair. Suggestions about the possible activity of Mms22 at this transition stage comes from genetic interaction data [36]. In these studies, *MMS22* clusters with *RTT109*, and *ASF1*, which are required for histone H3 acetylation [37]. These results suggest that Mms22, in association with Rtt109, and Asf1 are recruited to the sites of DNA lesions to modify their chromatin structure, perhaps facilitating DNA resection and recruitment of downstream-acting repair proteins such as Rad52. Recruitment of Rad52 in the form of foci depends on the removal Mms22 from DNA by the proteasome.

Taken together, we show that Mms22 is a proteasome target that links nuclear proteasomal activity and DSB repair. We believe that the CIN phenotype and impaired DNA repair caused by proteasome dysfunction can, in part, be attributed to the specific accumulation of Mms22. This idea is further supported by the observation that accumulation of Mms22 sensitizes the cells to DNA damaging agents, and results in CIN (Figure 2SA and Figure 3SD–3SF), and by previous studies showing that mutants in genes that play roles in DSB repair cause CIN phenotype in yeast and mammalian cells [31],[46]. It is likely that additional proteasomal targets important for genome stability await discovery. The mechanism of regulation of Mms22 may serve as a paradigm to understand how these additional proteins are regulated by the proteasome.

## Materials and Methods

### Yeast strains

Yeast strains that were used for the CTF screen are the result of backcrossing the haploid Ts strain (*MAT* **a** *ura3Δ0 leu2Δ0 his3Δ1 lys2Δ0 (*or *LYS2) met15Δ0 (*or *MET15) can1Δ::LEU2-MFA1pr::His3 yfeg-ts::URA3*), to the Donor strain SB1 (*MAT* **α** *ade2-101*::*NAT his3 ura3 lys2 can1*Δ *mfa1*Δ::*MFA1*pr-*HIS3* CFVII(*RAD2*.d)::*LYS2*), and selecting for a Lys^+^ spore clone (indicating the presence of CFVII(*RAD2*.d)::*LYS2*) resistant to ClonNAT (thus carrying the *ade2-101* ochre mutation) and Ura^+^ (*yfeg-ts::URA3*) [for more details see [19]]. Other strains that were used in this study are listed in Table S2. The following strains were generated by crossing the indicated strains (in brackets), and selecting for the appropriate spores: SB162-(SB158xTs944), SB163-(SB160xTs944), SB220-(SB158xTs670), SB223-(SB160xTs670), SB258-(SB256xSB148), SB259-(SB256xSB147), SB175-(Ts602Xsb132). Yeast strains used for DSB repair assay are isogenic derivatives of strain MK203 (*MATa-inc ura3::HOcs lys2::ura3::HOcs-inc ade3::GALHO ade2-1 leu2-3,112 his3-11,15 trp1-1 can1-100*) [29],[47], a derivative of W303. In SB276, a TEV protease consensus cleavage site was introduced at position 2801 of Mms22 in the strain K9127 [39] by a two-step gene replacement.

### Co-immunoprecipitations

Were preformed as previously described [48].

### Chromatin fractionation assay

Was performed as previously described [49]. Cells were grown to O.D600-0.5 in 50 ml culture. Samples were spun down in 50 ml conical tubes for 5 min, resuspended in 3 ml of 100 mM PIPES/KOH pH 9.4, 10 mM DTT, 0.1% Na-Azide, and incubated for 10 min at RT. Samples were then spun for 2 min. Supernatant was aspirated off, and samples were resuspended in 2 ml of 50 mM KPi, pH 7.4, 0.6M Sorbitol, 10 mM DTT, and transferred to 2 ml microfuge tubes. 10 ul aliquot was then diluted in 990 ul H2O in a cuvette. 4 ul of 20 mg/ml Zymolase T-100 was added for 10 min, in 37°C water bath (tubes were gently inverted every 2–3 minutes). After about 1 min, 10 ul aliquot was used to measure the O.D600 (for hypotonic lysis). The O.D of the 1∶100 dilutions after spheroplasting was less the 10% of the value before. From this point on everything was done in a cold room. Tubes were spun for 1 min, cells were then washed with 1 ml of 50 mM HEPES/KOH pH 7.5, 100 mM KCl, 2.5 mM MgCl2, 0.4M sorbitol. Tubes were spun for 1 min, and resuspended in equal pellet volume EB (around 80 ul). 1/40 volume 10% Triton X-100 (0.25% final, e.g. 4 ul for 160 ul suspension), was added and cells were incubated for 3 min for lysis on ice, (vortexed occasionally). This sample represents the whole cell extract (WCE). 20 ul sample was removed and 20 ul of SDS loading buffer was added (WCE). 100 ul EBX-S was prepared in separate microfuge tubes. 100 ul of whole cell extracts were laid onto the EBX-S, and microfuge tubes were spun for 10 min. The resulted fractions represent a white chromatin pellet (CHR), the clear sucrose layer, and above a yellow supernatant fraction (SUP). 20 ul of SDS loading buffer was added to 20 ul of the SUP fraction (SUP). The rest of supernatant and sucrose buffer were then aspirated. The chromatin pellet was resuspended in 100 ul EBX, and spun for 5 min. Supernatant was aspirated off, and chromatin pellet was resuspended again in 100 ul EBX. 20 ul sample was then removed and added to 20 ul of SDS loading buffer (CHR). EB: 50 mM HEPES/KOH pH 7.5, 100 mM KCl, 2.5 mM MgCl2, 1 mM DTT, 20 ug/ml leupeptin, 2 mM benzamidine, 2 ug/ml aprotinin, 0.2 mg/ml bacitracin, 2 ug/ml pepstatin A, 1 mM PMSF (add it just before use). EBX: EB +0.25% Triton X-100. EBX-S: EBX +30% Sucrose.

### Immunofluorescent labeling

Cells were plated onto sterilized glass coverslips so that they were 50% to 80% confluent on the following day. Subsequent to fixation for 5 min at 25°C with fresh 4.0% paraformaldehyde, cells were permeabilized with phosphate-buffered saline (PBS; pH 7.5) containing 0.5% Triton X-100 for 5 min. Cells were washed twice with PBS and subjected to sequential series of 30-min incubations with appropriate primary and secondary antibodies. Wash steps consisted of a single wash with PBS containing 0.1% Triton X-100 and two washes with PBS. The following primary antibodies were used: anti Psmd4 (Abcam ab20239), Psma1 (Abcam ab3325), anti 53BP1 (Abcam ab21083) and anti γH2AX (Abcam ab18311). Primary antibodies were recognized with appropriate mouse or rabbit secondary antibodies conjugated with either Alexa-fluor 488 or Cyanin-3 (Cy-3) (MolecularProbes, and the Jackson ImmunoResearch Laboratories respectively). Coverslips were mounted onto slides containing approximately 10 µl of a 90% glycerol-PBS–based medium containing 1 mg/mL parapheylenediamine and 0.5 µg/ml DAPI. Image acquisition and processing was preformed as detailed previously [50] using a Zeiss Axioplan 2 digital imaging microscope equipped with a ×63 (1.3 numerical aperture) and a x100 (1.4 numerical aperture) plan-apochromat oil-immersion lens, a Coolsnap HQ cooled charge-coupled device camera (Roper Scientific), and Metamorph imaging software (Universal Imaging Corp).

### The following procedures were performed as previously described [51], in brief:

#### Cell culture and siRNA transfection

HCT116 and Hela cells were cultured in McCoy's 5A and DMEM medium supplemented with 10% FBS in a 37°C humidified incubator containing 5% CO_2_. siRNA duplexes targeting *PSMA6, PSMD12, PSMD4* and *PSMA4* were purchased from Dharmacon. Transient transfection of HCT116 or Hela cells was performed using DharmaFECT 1 reagent as described by the manufacturer (Dharmacon).

#### Western blot analysis

To confirm protein knockdown and identify the most effective siRNA duplexes for each target, Western blots were conducted on proteins extracted from asynchronous and subconfluent cells 4 days post-transfection. Following protein transfer nitrocellulose membranes were blotted using the following Antibodies: anti PSMD4 (Abcam ab20239), anti PSMA4 (Abcam ab55625) and anti PSMA6 (Abcam ab2265). Alpha-tubulin mouse monoclonal antibody (Abcam ab7291) and GAPDH (Abcam ab9485) were used as a loading control.

#### Flow cytometry

Duplicate populations of asynchronous and subconfluent cells were harvested five days post-transfection, washed with PBS and permeablized with 70% Ethanol before PI-labeling. Cells were briefly sonicated to render a single cell suspension immediately before DNA content analysis.

#### Chromosome spreads and painting

To enrich for mitotic chromosomes, subconfluent cells were treated with KaryoMAX colcemid (0.1 µg/ml; Gibco) for 2 h before harvesting. Cells were trypsinized, pelleted (800 rpm, 5 min) and resuspended in hypotonic solution (75 mM KCl) for 5 min at room temperature. Cells were pelleted (5 min) and resuspended in freshly made methanol∶glacial acetic acid (3∶1), added drop-wise. Cells were repelleted (5 min), and resuspended in methanol∶glacial acetic acid as above. Two or three drops of suspended cells were applied to pre-cleaned blood smear glass slides.

#### CTF assay

CTF assay was performed as detailed previously [19],[31]. Each Ts allele was tested in a wide range of semi-and non-permissive temperatures (25°C, 30°C, 32°C, 34°C, and 37°C). Colony sectoring phenotypes were scored qualitatively as mild, intermediate and severe (indicated as 1, 2, and 3, respectively in Table S1).

#### cDNA isolation and RNA analysis

Was performed to verify the knock down of *PSMD12* as a result of siRNAi treatment. RNAs were extracted with a RNeasy Mini Kit (Qiagen). 350 ng of RNA were use for a first strand DNA synthesis (Invitrogen). cDNA was used as a template to detect the RNA levels of PSMD12 (Forward primer: TTTGTCTATTTGTAAGCACT/Reverse Primer: TTAAAAGATCCTTGTATTTG) and, GAPDH (Forward primer: TGACAACAGCCTCAAGATCA; Reverse Primer: CATCCACAGTCTTCTGGGTG).

#### DSB repair kinetic experiments

Were performed as previously described [29],[47]. In brief, the *S. cerevisiae* haploid test strain contains two copies of the *URA3* gene. One copy, located on chromosome *V*, carries the recognition site for the yeast HO site specific endonuclease (*ura3-HOcs*). The second copy, located on chromosome *II*, carries a similar site containing a single-base-pair mutation that prevents recognition by the HO endonuclease (*ura3-HOcs-inc*). In addition, the *ura3* alleles differ at two restriction sites, located to the left (*Bam*HI) and to the right (*Eco*RI) of the *HOcs-inc* insertion. These polymorphisms are used to follow the transfer of information between the chromosomes. In these strains, the HO-endonuclease gene is under the transcriptional control of the *GAL1* promoter. When cells are transferred to a galactose containing medium, the HO-endonuclease creates a single DSB. The broken chromosome is then repaired by a mechanism that copies the HOcs-inc information together with the flanking markers, resulting in a gene conversion event.

#### Media and growth conditions

*Saccharomyces cerevisiae* strains were grown at 30°C, unless specified otherwise. Standard YEP medium (1% yeast extract, 2% Bacto Peptone) supplemented with 3% glycerol (YEPGly), 2% galactose (YEPGal), or 2% dextrose (YEPD) was used for nonselective growth. 1.8% Bacto Agar was added for solid media.

#### DSB induction experiments

Single colonies were resuspended in rich YEPGly medium, grown to logarithmic phase, centrifuged and resuspended in YEPGly with and without 20 µM MG132 (CALBIOCHEM) for 2 hrs, followed by centrifugation and resuspension in YEPGal with and without 20 µM MG132. At timely intervals, samples were collected for FACS analysis, cells were inspected for cell cycle stage, and DNA was extracted and subjected to the different assays.

#### Southern blot analysis for DSB repair kinetics experiments

Was carried out as described previously [47]. The experiments shown in Figure 4 are reproducible, with a SD of about 10%. Rather than adding error bars to each of the data points presented, we show a representative example.

#### PCR assays

Portions (5 ng) of genomic DNA were amplified in each sample. Reactions were allowed to proceed to cycle 35. *Taq* polymerase was used in standard reaction conditions. The sequence of individual primers are available upon request.

#### Quantitation of results

Southern blot images were acquired by exposing the hybridized membrane to a standard X-ray film (FUJI) followed by scanning of the film to the computer.

Gel images were acquired by filming the EtBr stained gel under UV light.

Ethidium bromide-stained agarose gels and Southern blots were quantified using the GelQuant computer program (DNR Bio-imaging systems).

#### Genome-wide yeast-two-hybrid screens

*MMS22*, was cloned into pOBD2 as described in [52]. The Gal4p-Mms22p-DNA binding domain fusion protein was functional as determined by rescuing sensitivity of *mms22*Δ to 0.2M HU, 10 µg/ml camptothecin and 0.01% MMS (data not shown). Genome-wide two-hybrid screens were performed as described in [53]. Briefly, each screen was performed in duplicate, and positives that were identified twice were put into a mini-array for retest. Some reproducible positives were observed in many different screens with baits of unrelated function. These were considered as common false positives and were excluded from further analyses.

## Supporting Information

Figure S1 Western Blot analysis and RT–PCR to confirm human proteasomal subunits knockdown, and sensitivity of proteasomal CIN mutants to Hydroxyurea (HU). (A) Western Blot analysis and RT–PCR to confirm human proteasomal subunits knockdown. (B) Sensitivity of proteasomal CIN mutants to Hydroxyurea (HU). (A) siRNA-mediated knockdown of the indicated human proteasomal subunits in HCT116 cells examined by Western blot, or by RT–PCR (for *PSMD12*). Arrows at the top of each blot represent the siRNAs chosen for further analysis. A non-targeting siRNA control is also shown (NT siRNA). Anti-tubulin or GAPDH were used as loading controls. (B) Five-fold serial dilutions of the indicated proteasomal subunits mutants were spotted on YPD medium lacking or supplemented with 150 Mm of HU. Cells were incubated at 32°C and 34°C to find the semi-permissive temperature of each Ts mutant.
(0.99 MB TIF)

Figure S2 CIN phenotype of MK203 under proteasome inhibition; Mms22 and Rtt101 physically interact; the expression of Mms22 is regulated by the Ubiquitin-Proteasome System (UPS); Mms22 plays a role in DSB repair; ubiquitination of Mms22 is induced by DNA damage. (A) CIN phenotype of MK203 under proteasome inhibition. (B,C) Mms22 and Rtt101 physically interact. (D) The expression of Mms22 is regulated by the Ubiquitin-Proteasome System (UPS). (E) Mms22 plays a role in DSB repair. (F) Ubiquitination of Mms22 is induced by DNA damage. (A) a-like faker (ALF) assay reveals that MK203 cells under proteasome partial inhibition exhibit a higher level of CIN. ALF is based on the fact that the default mating type in yeast is *MAT*a. If the *MAT*α of MK203 lose the *MAT*α locus (due to the loss of chromosome III), they mate with a *MAT*α tester as *MAT*a, and are thus called “a-like fakers.” Two patches of a *MAT*α MK203 and control strains (wt Alpha, and *bim1*) were grown in the presence of galactose supplemented with MG132. These strains were replica plated on a lawn of *MAT*a tester strain. Growing colonies are the indication of the ability to mate with the tester strains. The ALF phenotype of MK203 is evident when compared to the wt control. (B) Yeast-two-hybrid interactions using the bait protein Mms22p. The mini array shown here represent re-tests of interactions that were identified in at least two genome wide screens. Each strain contains a different pOAD fusion protein. Positives interactors are indicated in yellow. A strain containing an empty pOAD was used as a negative control. MIG1 is a common false positive. (C) Mms22 and Rtt101 Co-ImmunoPrecipitation (IP). Doubly tagged Mms22-13Myc/Rtt101-3HA haploid strains and the singly tagged Mms22-13Myc control strain were subjected to IP with anti-Myc antibody. Whole Cell protein extracts (WCE), and IP samples, were subjected to immunoblotting with anti-Myc and anti-HA antibodies. In contrast to the single tagged control, Mms22-13Myc co-IPed with Rtt101-3HA. (D) Cyclohexamide chase experiments in cells expressing Mms22-HA. 100 µg/ml of Cyclohexamide was added to logarithmically growing samples (t-0) together with either DMSO control (left), 20 mM MG132 (middle) or DMSO in *rtt101* deletion background (right). Samples were collected at timely intervals, and Western blot analysis was used to detect the levels of Mmss22-HA. Pgk1 was used as a loading control. (E) PCR analysis of gene conversion product formation kinetics in *mms22* cells, and DMSO control. Treated cells show delayed gene conversion product formation, as apparent from the quantification graph (bottom). (F) Ubiquitination of Mms22 is induced by DNA damage in a *RTT101* dependent manner. Cells carrying 3HA*-*tagged Mms22 and 3Myc-Ubi (or a non tagged Ubi control), were grown in the presence of 20 mM MG132, and 0.025% MMS, and subjected to IP. After electrophoresis, the precipitated Mms22-HA protein was blotted to a membrane and immunobloted with an anti-HA antibody. Black arrow labels Mms22-3HA. Red or black stars represent Mms22-HA modified with the endogenous Ubi or Ubi-3Myc respectively, as revealed by successive immunoblotting with Anti-myc antibody. A red arrow labels the mono-ubiquitinated form of Mms22-HA.
(0.96 MB TIF)

Figure S3 Mms22 is recruited to chromatin upon DNA damage in a *RTT109* dependent manner; recruitment of Rpn5 to chromatin upon DNA damage; A control for the experiment described in Figure 5D; accumulation of Mms22 sensitizes the cells to DNA damaging agents, and results in CIN. (A) Mms22 is recruited to chromatin upon DNA damage in a *RTT109* dependent manner. (B) Recruitment of Rpn5 to chromatin upon DNA damage. (C) A control for the experiment described in Figure 5D. (D–F) Accumulation of Mms22 sensitizes the cells to DNA damaging agents, and results in CIN. (A) Experimental details are as in Figure 5C. Cell extracts (WCE) were separated into supernatant (SU) and chromatin (CH) fractions. Mms22 was detected by immunoblotting. Anti Carboxy peptidase-Y (CPY), and Anti Acetylated Histon H4 (AcH4) served as a SU and CH fractions controls respectively. (B) Experimental details are as in Figure 5C. (C) MMS treatment and not the prolonged exposure to MG132 treatment led to G_2_/M the accumulation and recruitment of Mms22 to chromatin. Cells were synchronized to G_1_ (5D#1) and released from the arrest in the presence of 20Mm MG132. A sample was collected at a time point similar to the sample shown in Figure 5D-5. FACs analysis and chromatin fractionation assay clearly show that cells exposed to MG132 only continued cycling normally, and Mms22 was mainly present at the SUP fraction in contrast to samples from a similar time point that was first exposed to MMS+MG132 (Figure 5D-5). (D) a-like faker (ALF) assay reveals that over expression (OE) of Mms22 results in Chromosomal instability. ALF was performed as described in Figure S2A. Two patches of a *MAT*α cells OE Mms22, and control strains (wt Alpha, and *bim1*) were replica plated on a lawn of *MAT* **a** tester strain. Growing colonies are the indication of the ability to mate with the tester strains. The ALF phenotype of OE Mms22 is evident when compared to the wt control. (E, F) Overexpression of Mms22 results in growth defects in the presence of DNA damaging agents. Serial dilutions of the indicated strains were spotted on the indicated media (E) Overexpression (OE) of Mms22 affects growth upon induction of a specific DSB. Plating on galactose induced a DSB at the HOcs, together with the OE of the Mms22 protein. (F) Mms22 OE results in growth defect on media supplemented with the indicated drugs. Overexpression of Mms22 in a *rtt101Δ* mutant shows the same sensitivity as the single *rtt101Δ* mutant, as expected from an epistatic interaction.
(1.88 MB TIF)

Table S1 Genes identified through ts mutants that affect CIN, quantification of the CIN phenotype, and E-value of their human homolog.
(0.07 MB DOC)

Table S2 Yeast strains used in this study.
(0.06 MB DOC)
