# Supplementary figures and images for: Proteasome Nuclear Activity Affects Chromosome Stability by Controlling the Turnover of Mms22, a Protein Important for DNA Repair
Source: PLoS Genet. 2010 Feb 19;6(2):e1000852. doi: 10.1371/journal.pgen.1000852 (PMC2824753; doi:10.1371/journal.pgen.1000852)

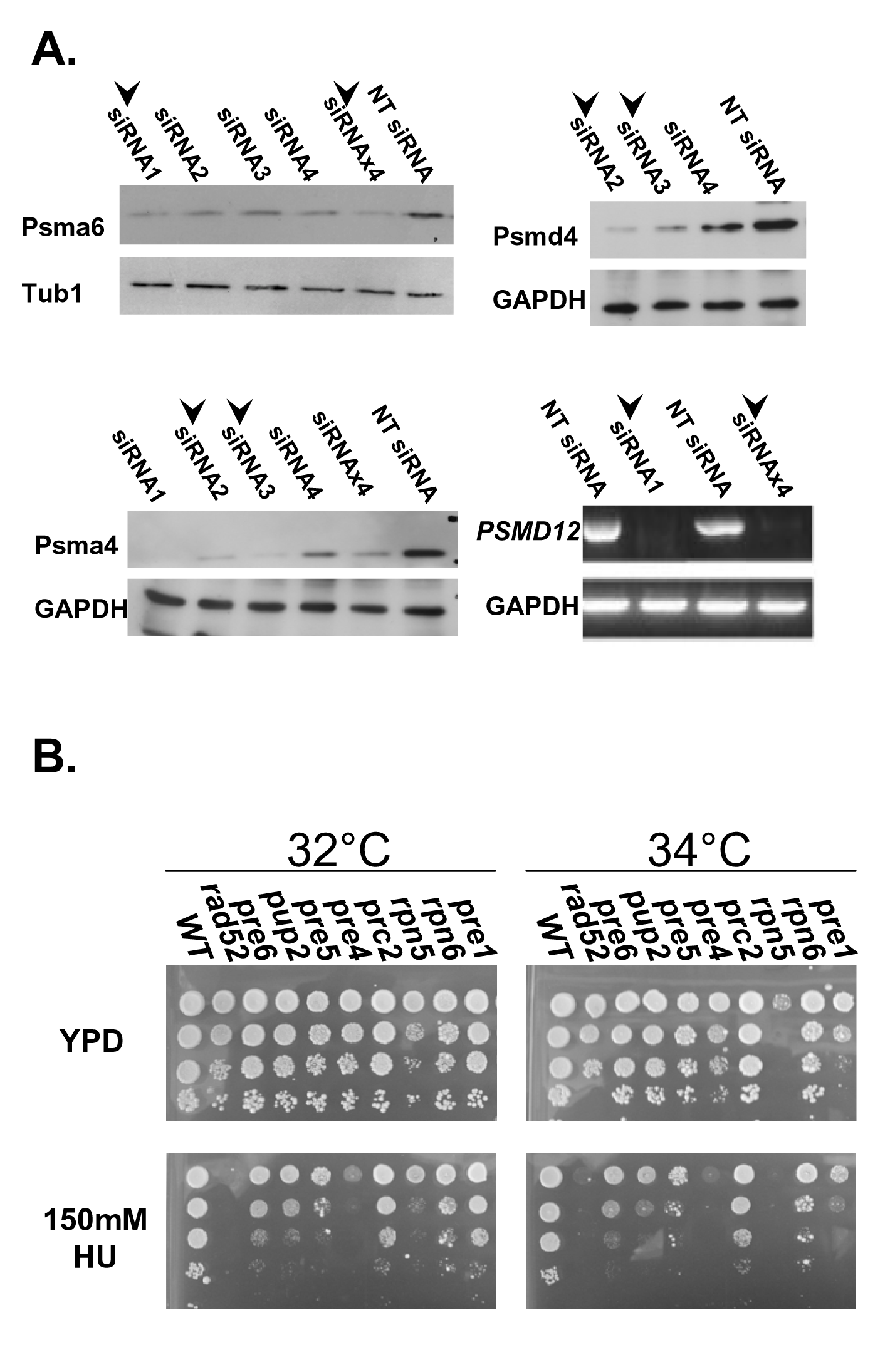

Supplement: Figure S1 — Western Blot analysis and RT–PCR to confirm human proteasomal subunits knockdown, and sensitivity of proteasomal CIN mutants to Hydroxyurea (HU). (A) Western Blot analysis and RT–PCR to confirm human proteasomal subunits knockdown. (B) Sensitivity of proteasomal CIN mutants to Hydroxyurea (HU). (A) siRNA-mediated knockdown of the indicated human proteasomal subunits in HCT116 cells examined by Western blot, or by RT–PCR (for PSMD12). Arrows at the top of each blot represent the siRNAs chosen for further analysis. A non-targeting siRNA control is also shown (NT siRNA). Anti-tubulin or GAPDH were used as loading controls. (B) Five-fold serial dilutions of the indicated proteasomal subunits mutants were spotted on YPD medium lacking or supplemented with 150 Mm of HU. Cells were incubated at 32°C and 34°C to find the semi-permissive temperature of each Ts mutant. (0.99 MB TIF) [file pgen.1000852.s001.tif]

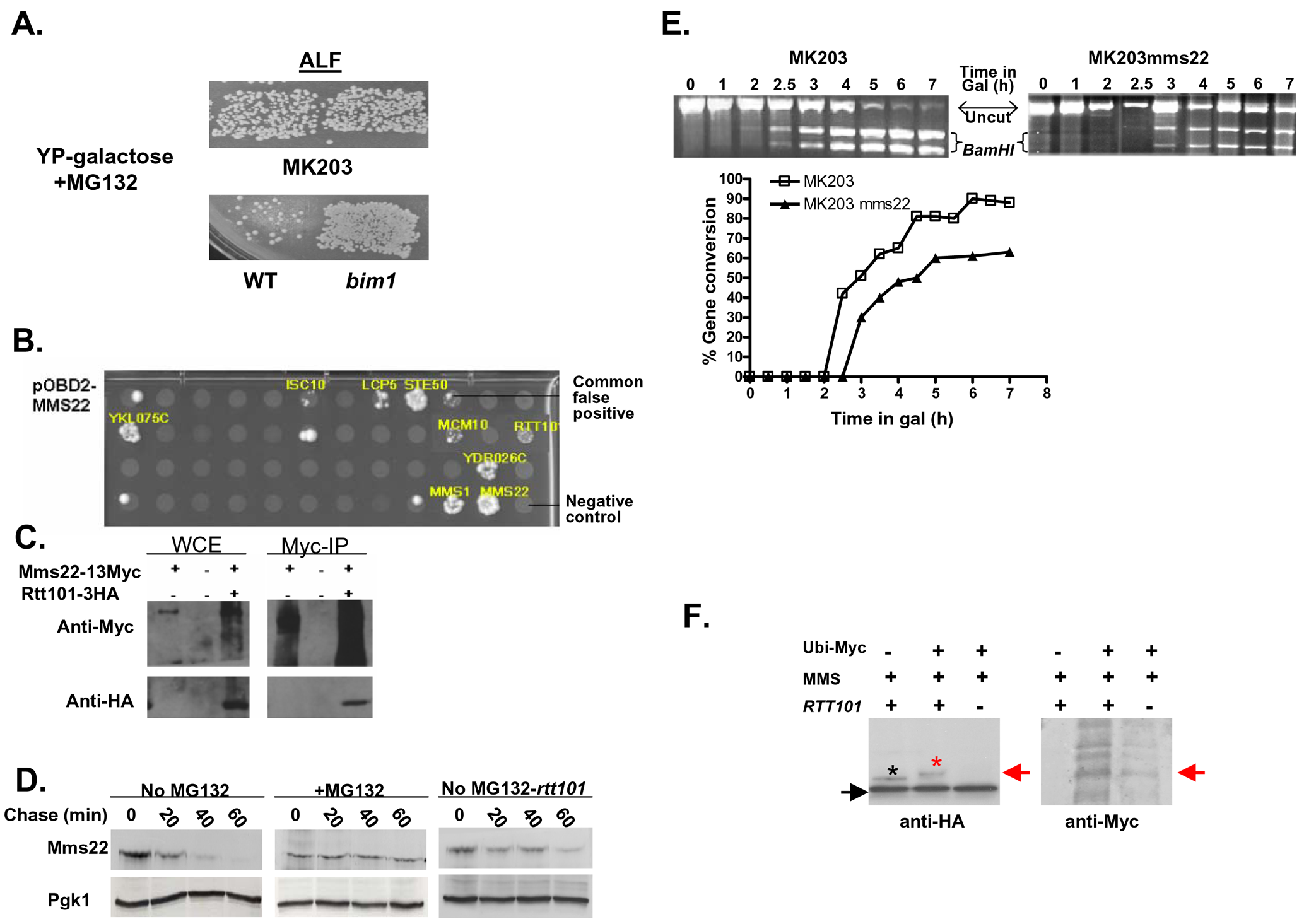

Supplement: Figure S2 — CIN phenotype of MK203 under proteasome inhibition; Mms22 and Rtt101 physically interact; the expression of Mms22 is regulated by the Ubiquitin-Proteasome System (UPS); Mms22 plays a role in DSB repair; ubiquitination of Mms22 is induced by DNA damage. (A) CIN phenotype of MK203 under proteasome inhibition. (B,C) Mms22 and Rtt101 physically interact. (D) The expression of Mms22 is regulated by the Ubiquitin-Proteasome System (UPS). (E) Mms22 plays a role in DSB repair. (F) Ubiquitination of Mms22 is induced by DNA damage. (A) a-like faker (ALF) assay reveals that MK203 cells under proteasome partial inhibition exhibit a higher level of CIN. ALF is based on the fact that the default mating type in yeast is MATa. If the MATα of MK203 lose the MATα locus (due to the loss of chromosome III), they mate with a MATα tester as MATa, and are thus called “a-like fakers.” Two patches of a MATα MK203 and control strains (wt Alpha, and bim1) were grown in the presence of galactose supplemented with MG132. These strains were replica plated on a lawn of MATa tester strain. Growing colonies are the indication of the ability to mate with the tester strains. The ALF phenotype of MK203 is evident when compared to the wt control. (B) Yeast-two-hybrid interactions using the bait protein Mms22p. The mini array shown here represent re-tests of interactions that were identified in at least two genome wide screens. Each strain contains a different pOAD fusion protein. Positives interactors are indicated in yellow. A strain containing an empty pOAD was used as a negative control. MIG1 is a common false positive. (C) Mms22 and Rtt101 Co-ImmunoPrecipitation (IP). Doubly tagged Mms22-13Myc/Rtt101-3HA haploid strains and the singly tagged Mms22-13Myc control strain were subjected to IP with anti-Myc antibody. Whole Cell protein extracts (WCE), and IP samples, were subjected to immunoblotting with anti-Myc and anti-HA antibodies. In contrast to the single tagged control, Mms22-13Myc co-IPed with [file pgen.1000852.s002.tif]

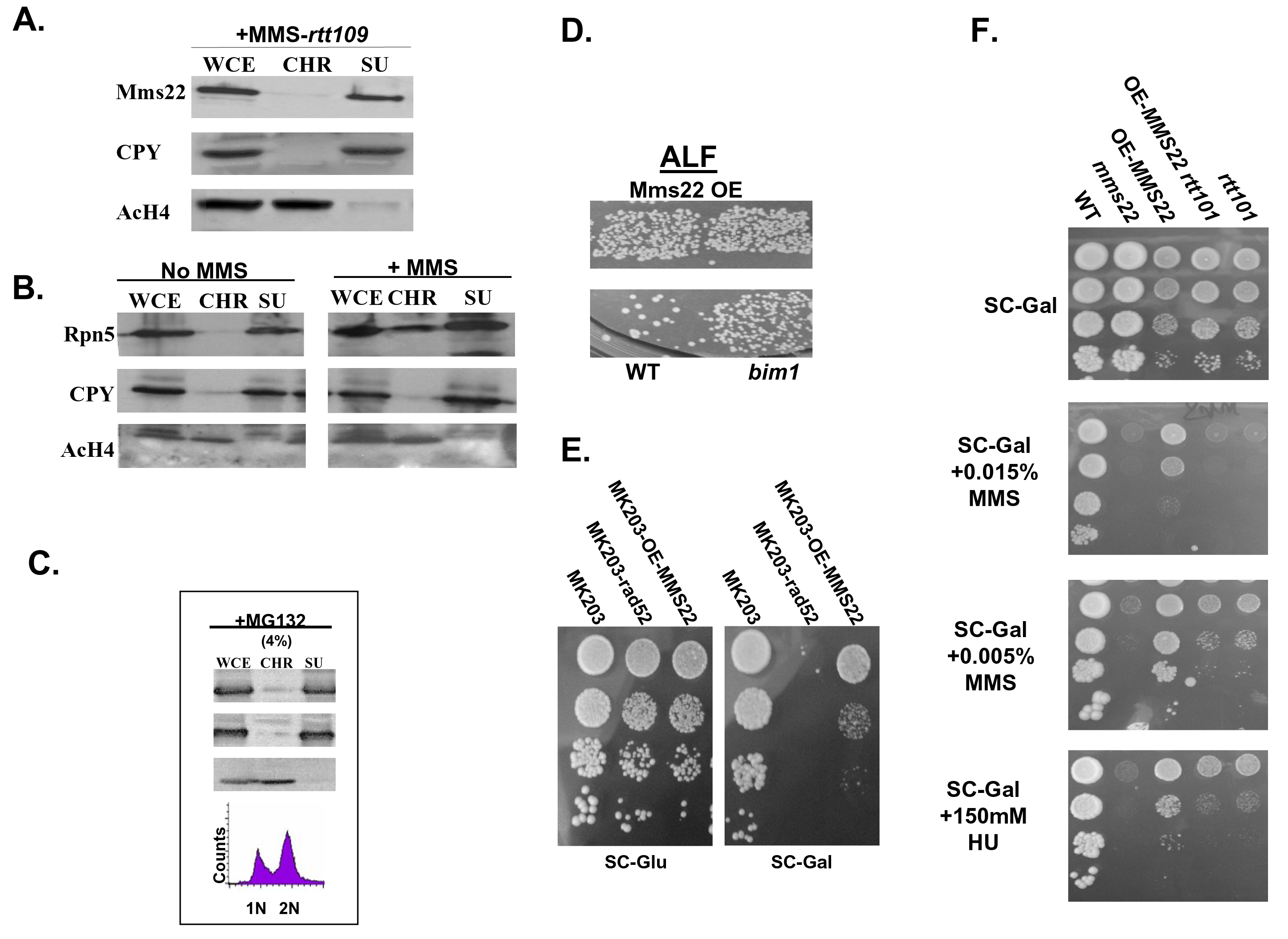

Supplement: Figure S3 — Mms22 is recruited to chromatin upon DNA damage in a RTT109 dependent manner; recruitment of Rpn5 to chromatin upon DNA damage; A control for the experiment described in Figure 5D; accumulation of Mms22 sensitizes the cells to DNA damaging agents, and results in CIN. (A) Mms22 is recruited to chromatin upon DNA damage in a RTT109 dependent manner. (B) Recruitment of Rpn5 to chromatin upon DNA damage. (C) A control for the experiment described in Figure 5D. (D–F) Accumulation of Mms22 sensitizes the cells to DNA damaging agents, and results in CIN. (A) Experimental details are as in Figure 5C. Cell extracts (WCE) were separated into supernatant (SU) and chromatin (CH) fractions. Mms22 was detected by immunoblotting. Anti Carboxy peptidase-Y (CPY), and Anti Acetylated Histon H4 (AcH4) served as a SU and CH fractions controls respectively. (B) Experimental details are as in Figure 5C. (C) MMS treatment and not the prolonged exposure to MG132 treatment led to G2/M the accumulation and recruitment of Mms22 to chromatin. Cells were synchronized to G1 (5D#1) and released from the arrest in the presence of 20Mm MG132. A sample was collected at a time point similar to the sample shown in Figure 5D-5. FACs analysis and chromatin fractionation assay clearly show that cells exposed to MG132 only continued cycling normally, and Mms22 was mainly present at the SUP fraction in contrast to samples from a similar time point that was first exposed to MMS+MG132 (Figure 5D-5). (D) a-like faker (ALF) assay reveals that over expression (OE) of Mms22 results in Chromosomal instability. ALF was performed as described in Figure S2A. Two patches of a MATα cells OE Mms22, and control strains (wt Alpha, and bim1) were replica plated on a lawn of MAT a tester strain. Growing colonies are the indication of the ability to mate with the tester strains. The ALF phenotype of OE Mms22 is evident when compared to the wt control. (E, F) Overexpression of Mms22 results in growth defects in the presence [file pgen.1000852.s003.tif]
