# Supplementary material for: Proteasome Nuclear Activity Affects Chromosome Stability by Controlling the Turnover of Mms22, a Protein Important for DNA Repair
Source: PLoS Genet. 2010 Feb 19;6(2):e1000852. doi: 10.1371/journal.pgen.1000852 (PMC2824753; doi:10.1371/journal.pgen.1000852)
Supplement: Table S1 — Genes identified through ts mutants that affect CIN, quantification of the CIN phenotype, and E-value of their human homolog. (0.07 MB DOC) [file pgen.1000852.s004.doc]

| **Table S1. Genes Identified through ts Mutants that Affect CIN, Quantification of the CIN Phenotype, and E-Value of their Human homolog** | | | | |
| --- | --- | --- | --- | --- |
| Yeast  Gene Name | Human  Homolog | Description | CTF Score | E-Value of  Human homolog |
| *PRC2* | *PSMA6* | Proteasome | 2 | 1.00E-62 |
| *PRE4* | *PSMB4* | Proteasome | 1 or 2 | 4.00E-47 |
| *PRE6* | *PSMA7* | Proteasome | 2 or 3 | 3.00E-71 |
| *PUP2* | *PSMA5* | Proteasome | 2 | 5.00E-69 |
| *PRE5* | *PSMA1* | Proteasome | 1 | 3.00E-64 |
| *RPN6* | *PSMD11* | Proteasome | 2 | 2.00E-75 |
| *RPN5* | *PSMD12* | Protesome | 2 | 1.00E-85 |
| *PBN1* | ***/*** | Post translation modification | 3 | No homolog |
| *BIG1* | */* | Post translation modification | 2 | No homolog |
| *UPG1* | *UGP2* | Post translation modification | 2 | 1.00E-261 |
| *GPL17* | *PIGS* | Post translation modification | 3 | 1.00E-10 |
| *GPL10* | *PIGB* | Post translation modification | 1 to 2 | 1.00E-33 |
| *MSL5* | *SF1* | Splicing | 3 | 4.00E-41 |
| *CWC2* | *RBM22* | Splicing | 3 | No homolog |
| *AAR2* | *C20ORF4* | Splicing | 3 | 2.00E-05 |
| *HSH155* | *SF3B1* | Splicing | 1 | 1.00E-261 |
| *SPP382* | *TFIP11* | Splicing | 1 to 2 | No homolog |
| *PSF3* | *GINS3* | DNA replication | 3 | No homolog |
| *ORC1* | *ORC1L* | DNA replication | 3 | 2.00E-41 |
| *ORC6* | */* | DNA replication | 2 | No homolog |
| *POL30* | *PCNA* | DNA replication | 3 | 7.00E-46 |
| *RPB5* | *POLR2E* | Transcription | 3 | 8.00E-41 |
| *SSL1* | *GTF2H2* | Transcription | 2 | 4.00E-73 |
| *MCM1* | *SRF* | Transcription | 2 | 8.00E-14 |
| *UTP5* | *WDR43* | rRNA processing | 3 | 5.00E-10 |
| *PWP1* | *PWP1* | rRNA processing | 3 | 1.00E-50 |
| *TRM5* | *TRMT5* | rRNA processing | 2 | 3.00E-58 |
| *RLI1* | *ABCE1* | Ribosome biogenesis | 2 | 1.00E-261 |
| *RLP7* | *RPL7* | Ribosome biogenesis | 3 | 8.00E-19 |
| *GRS1* | *GARS* | tRNA synthesis | 3 | 1.00E-159 |
| *FRS1* | *FARSB* | tRNA synthesis | 3 | 1.00E-141 |
| *GCD7* | *ELF2B2* | Translation | 1 | 5.00E-39 |
| *GCD1* | *ELF2B3* | Translation | 2 to 3 | 4.00E-11 |
| *STS1* | */* | Protein degradation | 1 | No homolog |
| *TRS130* | *TMEM1* | Protein trafficing | 3 | 6.00E-05 |
| *PRO3* | *PYCR1* | proline biogenesis | 3 | 1.00E-19 |
| *APC4* | */* | Subunit of APC/C | 2 | No homolog |
| *SPC19* | */* | Kinetochore | 1 | No homolog |
| *SPC98* | *TUBGCP3* | Microtubule-nucliating complex | 2 | 3.00E-29 |
| *RVB2* | *RUVBL2* | Chromatin remodeling | 3 | 1.00E-149 |
