# Supplementary material for: Proteasome Nuclear Activity Affects Chromosome Stability by Controlling the Turnover of Mms22, a Protein Important for DNA Repair
Source: PLoS Genet. 2010 Feb 19;6(2):e1000852. doi: 10.1371/journal.pgen.1000852 (PMC2824753; doi:10.1371/journal.pgen.1000852)
Supplement: Table S2 — Yeast strains used in this study. (0.06 MB DOC) [file pgen.1000852.s005.doc]

| **Table S2. Yeast strains used in this study** | | |
| --- | --- | --- |
| Strain | Genotype | Reference |
| SB132 | *MAT***a** *ura3Δ0 leu2Δo his3Δ1 met15Δ0 RPN5*-*GFP*-*KmX* | This Study |
| SB240 | *MAT***α** *ura3Δ0 leu2Δo his3Δ1 lys2Δ0 met15Δ0 PUP2*-*GFP*-*KmX* | This Study |
| SB147 | *MAT***a** *ura3Δ0 leu2Δo his3Δ1 met15Δ0 KmX-GAL1-GFP-RPN5* | This Study |
| SB148 | *MAT***a** *ura3Δ0 leu2Δo his3Δ1 met15Δ0 KmX-GAL1-GFP-rpn-*Ts*-URA3* | This Study |
| SB241 | *MAT***a** *ura3Δ0 leu2Δo his3Δ1 met15Δ0 pup2-*Ts-*GFP*-KmX-*URA3* | This Study |
| SB158 | *MAT***a** *leu2-3, 112 trp1-1 can1-100 ura3-1 ade2-1 his311,15 bar1::LEU2 pep4::TRP1 RPN11-GFP-HIS3* | T. Mayor |
| SB160 | *MAT***a** *leu2-3, 112 trp1-1 can1-100 ura3-1 ade2-1 his311,15 bar1::LEU2 pep4::TRP1 PRE6-GFP-HIS3* | T. Mayor |
| SB162 | *MAT***a** *RPN11-GFP-HIS3 rpn5-Ts-URA3* | This Study |
| SB163 | *MAT***a** *PRE6-GFP-HIS3 rpn5-Ts-URA3* | This Study |
| SB220 | *MAT***a** *RPN11-GFP-HIS3 pup2-Ts-URA3* | This Study |
| SB223 | *MAT***a** *RRE6-GFP-HIS3 pup2-Ts-URA3* | This Study |
| SB258 | *STS1-TAP-HISMX6* *KmX-GAL1-GFP-rpn-*Ts*-URA3* | This Study |
| SB259 | *STS1-TAP-HIS3MX6 KmX-GAL1-GFP-RPN5* | This Study |
| MK203 | *MAT*α*-inc ade2 ade3::GALHO ura3::HOcs leu2-3,112 his3-11,13 trp1-1 lys2::ura3::HOcs-inc(RB)* | {Aylon, 2003 #131} |
| MK203  *rad52* | *MAT*α*-inc ade2 ade3::GALHO ura3::HOcs leu2-3,112 his3-11,13 trp1-1 lys2::ura3::HOcs-inc(RB) rad52::LEU2* | {Aylon, 2003 #131} |
| SB238 | *MAT*a*-inc ade2 ade3::GALHO ura3::HOcs leu2-3,112 his3-11,13 trp1-1 lys2::ura3::HOcs-inc(RB)pdr5::hygB* | This Study |
| YSJ119 | *ho hmlΔ::ADE1 matαΔ::hisG hmrΔ::ADE1 leu2::KAN ade3::GAL1::HO ade1 lys5 ura3-52 trp1 can1::leu2-HOcs* chromosome *III*:*LEU2* (at position 41400 from the left end of chromosome *III*) | {Lydeard, 2007 #135} |
| SB228 | *ho hmlΔ::ADE1 matαΔ::hisG hmrΔ::ADE1 leu2::KAN ade3::GAL1::HO ade1 lys5 ura3-52 trp1 can1::leu2-HOcs* chromosome *III*:*LEU2* (at position 41400 from the left end of chromosome *III*) *pdr5::hygB* | This Study |
| YKY64 | *MATa ura3-52 trp1*Δ*-63 his3*Δ*-200 leu2*Δ*-1 ade2-101 lys2-801 mms22*Δ*::HIS3* | This Study |
| YKY527 | *MATa ura3-52 trp1*Δ*-63 his3*Δ*-200 leu2*Δ*-1 ade2-101 lys2-801 HIS3-pGAL-3HA-MMS22* | This Study |
| SB267 | *MATa ura3-52 trp1*Δ*-63 his3*Δ*-200 leu2*Δ*-1 ade2-101 lys2-801 MMS22-3HA-HIS3* | This Study |
| YKY782 | *MATa ura3-52 trp1*Δ*-63 his3*Δ*-200 leu2*Δ*-1 ade2-101 lys2-801 HIS3-pGAL-3HA-MMS22 rtt101*Δ*::TRP1* | This Study |
| Ts602 | *MAT***a** *ura3Δ0 leu2Δ0 his3Δ1 lys2Δ0 met15Δ0* or *MET15)* *sts1::URA3* | This Study |
| SB175 | *MAT***a** *ura3Δ0 leu2Δ0 his3Δ1 lys2Δ0 met15Δ0* or *MET15)* *sts1-Ts::URA3 RPN5-GFP-KmX* | This Study |
| SB256 | *MATa his3Δ1 leu2Δ0 met15Δ0 ura3Δ0 STS1-*TAP*-HIS3MX6* | OpenBiosystems |
| Ts944 | *MAT***α** *ura3Δ0 leu2Δ0 his3Δ1 lys2Δ0( met15Δ0* or *MET15)* *rpn5-*Ts*::URA3* | This Study |
| Ts670 | *MAT***α** *ura3Δ0 leu2Δ0 his3Δ1 lys2Δ0( met15Δ0* or *MET15)* *pup2-*Ts*::URA3* | This Study |
| SB275 | *MAT***a** *MMS22-TEV2801 GAL-NLS-myc9-TEV-NLS2x10* | This Study |
